# "We call them miracle babies": How health care providers understand neonatal near-misses at three teaching hospitals in Ghana

**DOI:** 10.1371/journal.pone.0198169

**Published:** 2018-05-30

**Authors:** April J. Bell, Lynette V. Wynn, Ashura Bakari, Samuel A. Oppong, Jessica Youngblood, Zelda Arku, Yemah Bockarie, Joseph Adu, Priscilla Wobil, Gyikua Plange-Rhule, Bamenla Goka, Richard M. Adanu, Cheryl A. Moyer

**Affiliations:** 1 Department of Environmental Health Sciences, University of Michigan School of Public Health, Ann Arbor, MI, United States of America; 2 Global REACH, University of Michigan Medical School, Ann Arbor, MI, United States of America; 3 Medical School, University of Michigan, Ann Arbor, MI, United States of America; 4 Child Health Department, Suntresu Hospital, Ghana Health Service, Kumasi, Ghana; 5 Department of Obstetrics and Gynaecology, School of Medicine and Dentistry, University of Ghana, Accra, Ghana; 6 Neonatal Intensive Care Unit, Cape Coast Teaching Hospital, Cape Coast, Ghana; 7 Department of Obstetrics and Gynaecology, Cape Coast Teaching Hospital, Cape Coast, Ghana; 8 Child Health Department, Komfo Anokye Teaching Hospital, Kumasi, Ghana; 9 Department of Child Health, School of Medicine and Dentistry, University of Ghana, Accra, Ghana; 10 School of Public Health, University of Ghana, Legon, Ghana; 11 Department of Learning Health Sciences, University of Michigan, Ann Arbor, MI, United States of America; 12 Department of Obstetrics and Gynecology, University of Michigan, Ann Arbor, MI, United States of America; Instituto de Nutricion de Centroamerica y Panama, GUATEMALA

## Abstract

Neonatal mortality is a significant problem in many low-resource countries, yet for every death there are many more newborns who suffer a life-threatening complication but survive. These “near-misses” are not well defined, nor are they well understood. This study sought to explore how health care providers at three tertiary care centers in Ghana (each with neonatal intensive care units (NICUs)) understand the term “near-miss.” Eighteen providers from the NICUs at three teaching hospitals in Ghana (Korle Bu Teaching Hospital in Accra, Komfo Anokye Teaching Hospital in Kumasi, and Cape Coast Teaching Hospital in Cape Coast) were interviewed in depth regarding their perceptions of neonatal morbidity, mortality, and survival. Near the end of the interview, they were specifically asked what they understood the term “near-miss” to mean. Participants included nurses and physicians at various levels and with varying years of practice (mean years of practice = 9.33, mean years in NICU = 3.66). Results indicate that the concept of “near-misses” is not universally understood, and providers differ on whether a baby is a near-miss or not. Providers disagreed on the utility of a near-miss classification for clinical practice, with some suggesting it would be helpful to draw their attention to those at highest risk of dying, with others suggesting that the acuity of illness in a NICU means any baby could become a ‘near-miss’ at any moment. Further efforts are needed to standardize the definitions of neonatal near-misses, including developing criteria that are able to be assessed in a low-resource setting. In addition, further research is warranted to determine the practical implications of using a near miss tool in the process of providing care in a resource-limited setting and whether it might be best reserved as a retrospective indicator of overall quality of care provided.

## Introduction

Nearly 3 million newborn babies die every year, and one third of these deaths occur in the first 24 hours after delivery[1,2]. The most common causes of neonatal deaths (or those deaths that occur within 28 days of birth) are often preventable, including birth asphyxia, neonatal sepsis, and complications from prematurity.[3] The vast majority of these deaths occur in low-resource settings. In sub-Saharan Africa alone, newborn mortality accounts for 44% of all deaths of children under age 5.[4]

While neonatal mortality is a significant problem in LMICs, for every death there are many more newborns who narrowly miss dying and may suffer long-term consequences as a result. Neonatal “near misses” are newborns who survive a life-threatening condition.[5] One study conducted in Brazil found that neonatal near misses outnumber neonatal deaths three to one[6], meaning that for every neonatal death there were three infants who nearly died but didn’t. Other studies suggest neonatal near-misses could outnumber deaths five or six to one.[7]

Unlike maternal near misses, however, there is no standard definition and validated tool to identify neonatal near misses. Maternal near-misses have been studied repeatedly, such that there is a World Health Organization Maternal Near-Miss Tool that is commonly used to conduct near-miss assessments.[8] Yet assessments of neonatal near-misses have varied in their criteria for inclusion (e.g. symptoms, interventions, organ system dysfunction) and their window of observation (3 days, 7 days, 28 days).[7] Despite this variability, assessment of neonatal near-misses is becoming increasingly common, and this was illustrated in a recent systematic review of the literature by Santos and colleagues.[7]

Despite the increasing use of neonatal near-miss assessments, it is not clear how clinicians in neonatal care units in low-resource settings might view this classification. Would knowing a newborn counted as a near-miss have any effect on subsequent care provided? Is ‘near-miss’ as a classification useful for clinicians? Or is it better reserved as an indicator of overall quality of care, given that reduced mortality makes it harder to see improvements in relatively rare events? This study sought to explore how health care providers understand the term ‘near-miss’, focusing on three neonatal intensive care units at three teaching hospitals across Ghana.

## Methods

### Design

This study was an exploratory, qualitative study, nested within a larger, prospective study of maternal and neonatal near-misses at three tertiary care centres in Ghana. Data presented here reflect qualitative data collected from a subset of the participants included in the larger study.

### Setting

Data were collected from April through August 2015 at the Cape Coast Teaching Hospital (CCTH), Komfo-Anokye Teaching Hospital (KATH), and Korle Bu Teaching Hospital (KBTH) in southern Ghana.

CCTH is located in Cape Coast (population approximately 170,000), which is the smallest district in Ghana in terms of land size, the poorest in Central Region, and the fourth poorest region in Ghana. [9,10] It serves as the main referral hospital for most of the rural Central and parts of Western regions of Ghana. The hospital also serves as the teaching hospital of the University of Cape Coast, School of Medical Sciences (UCC-SMS). It has 369 beds and conducts approximately 3600 deliveries per year. The neonatal intensive care unit has approximately 782 admissions each year.

KATH is a teaching hospital affiliated with the Kwame Nkrumah University of Science and Technology School of Medical Sciences (KNUST-SMS) located in Kumasi, the second largest city in Ghana, with a population of about 2 million.[11] KATH serves as a referral center for most of the northern region, central, western, eastern and part of the Volta regions.[12] KATH oversees about 11,000 deliveries annually, and the KATH mother and baby unit, which provides care for ill babies from 0–60 days, has approximately 4500 admissions each year.[13]

KBTH, the teaching hospital associated with the University of Ghana School of Medicine and Dentistry is located in the capital city of Accra, with a population of about 4 million.[11] It is the largest tertiary referral hospital in Ghana and serves as the major referral center for most of southern Ghana. KBTH has 2400 beds and oversees approximately 11,000 deliveries annually. About 2500 newborns are admitted to the neonatal intensive care unit (NICU) annually. (Unpublished data)

### Participants

Selected health care providers who work in the neonatal intensive care units (NICUs) at KATH, KBTH, and CCTH were asked to participate in this research. Eligible participants were physicians or nurses with experience working in the NICU who were working between April and August 2015. No limit on duration of experience in the NICU was specified. At each site, 3 doctors and 3 nurses were selected, each with a different level of experience (e.g. house officer, medical officer, senior medical officer, resident, specialist, consultant, staff nurse, staff enrolled nurse). Participants were selected by site facilitators based on level of experience and availability. A total of 18 health care providers gave consent for participation, representing 11.8% of the 152 total providers on the employment roster across all three hospitals.

### Interviewers

Indepth interviews were conducted by a trained research assistant with several years’ experience in qualitative research methodology. Interviews, which typically lasted between 30 and 60 minutes, were conducted in English and transcribed verbatim.

### Interview guide

A brief questionnaire was developed to assess demographic information and training history for participating healthcare workers. The in-depth interview guide was developed using an iterative process amongst the investigators, eight of whom are clinicians (AB, SAO, YB, JA, PW, GPR, BG, RMA) who have experience in the study sites. The tool was pilot tested among members of the target population and revised prior to study initiation. Table 1 illustrates some of the questions providers were asked.

**Table 1:** Excerpts from the semi-structured interview tool used with providers.

Excerpted Questions From The Provider Interview Tool
• Some babies had severe complications. Some you may have struggled with and they died, some of them you may have struggled with and they lived. Some died no matter what you did. Some lived when you thought that they would not have. What do you think was the difference between those who lived and those who died? • Can you tell me about your experiences with the babies who lived when you thought they were going to die? What types of diseases or conditions did these babies have? • Are mothers usually given special instructions for caring for babies that lived when they looked like they were going to die? Do mothers usually understand these instructions? Why or why not? Are there any cultural barriers to mothers following these instructions? • Have you ever received formal training on how to handle these cases (of babies that survived when you thought they would have died)? • Are you familiar with the term “near miss”? Please tell me what it means to you. • Do you think providers all have the same idea of what a ‘near miss’ is? • Is it [near miss] a distinction that is useful to you as a provider? Why or why not? • If you knew a baby was classified as having a “near-miss” event, do you think it would change how you managed that baby? • What challenges do you think that babies who experienced a near miss will face as they age? • How do you think the health care system will respond to their needs?

### Ethical clearance

Ethical clearance was obtained from the institutional review boards at the Kwame Nkrumah University of Science and Technology for KATH, University of Cape Coast for CCTH, University of Ghana for KBTH, and the University of Michigan.

Information about the objectives of the discussion and the purpose of the overall study were provided to each potential participant. Confidentiality with regard to their participation and anonymity with regard to their data were assured. Written informed consent was obtained from each participant. Permission to audio-record the interviews was obtained.

### Data collection

Research assistants approached providers during their time in the NICU, explained the study, and scheduled interviews at a time that was convenient for the provider. Interviews took place in a staff room in the pediatric ward and ranged from 30–60 minutes.

Demographic data were collected using the Qualtrics offline data collection application for Iphone before the start of each interview. (Qualtrics, Provo, Utah, USA)

Interviews were recorded on iGearPro Multifunctional Voice Recorder or via Smart record application on Iphone. Interviews were transcribed verbatim using Infinity Foot Control transcription pedal with Express Scribe software.

### Data analysis

Quantitative data on participant demographics were entered into Stata V 13.1 and analyzed for descriptive statistics such as mean, range, and distribution.

All qualitative interviews were read by at least two of the investigators (AB, JY, CM) and “in-vivo” coding was conducted to assist in the identification of main codes. In-vivo coding involves making written notes on hard copies of the transcripts and reviewing the notes together. This iterative process used in-vivo coding to develop a preliminary coding structure and supporting codebook. All transcripts were entered into NVivo 10.0, a qualitative software analysis package. Focused coding (using the initial coding structure as a guide) was conducted by 4 separate coders (AB, JY, ZA, CM). Coders held regular meetings to review and revise the codebook to reflect inclusion and exclusion criteria that may not have arisen previously.

## Results

Table 2 illustrates the demographics of the sample. Providers ranged in age from 25 to 53, with nurses being slightly older than physicians (average age of nurses was 38.2 versus 31.1 years for physicians). Providers averaged 9.3 years of practice, with nurses averaging 13.2 years and physicians averaging 5.4 years of practice. Time in the NICU also varied, with nurses averaging 6.3 years of NICU experience while physicians averaged slightly more than one year of NICU experience.

**Table 2:** Health care provider demographics.

	All Providers (N = 18)	Physicians* (N = 9)	Nurses** (N = 9)
Mean/Median (range) Age	34.67 / 33 (25–53)	31.1 / 30 (25–42)	38.2 / 37 (29–53)
Mean/Median (range) Years of practice	9.33 / 7 (0.9–25)	5.43 / 5 (0.9–14)	13.22 / 13 (4–25)
Mean/Median (range) Years at institution/hospital	7.19 / 4.5 (0.75–25)	3.39 / 2 (0.75–8)	11.0 / 12 (1–25)
Mean/Median (range) Years in NICU	3.66 / 1 (0.1–20)	1.02 / 0.25 (0.1–6.0)	6.31 / 4 (0.25–20)
N, % Male	3 (16.6%)	3 (33.3%)	0 (0.0)

*Senior specialist (1), junior specialist (2), medical officer (1), house officer (3), other (2)

**Senior nursing officer (6), junior nursing officer (1), staff nurse (1), other (1)

The concept of “near-misses” was not universally understood by healthcare providers, regardless of their level of training, and regardless of whether providers were doctors or nurses. Some providers said they had heard of near-misses before but couldn’t remember what they were, while others said they didn’t think providers understood what constituted a near-miss. Still others confused neonatal near-miss mortality with missed opportunities for immunizations.

“It’s a near-miss if you don’t immunize them before they go (home from the hospital).” (Female physician, 1 year experience, KATH, Kumasi)

At its most fundamental level, near-misses were described as “…if the baby that you think would die hasn’t died and the baby survives…. That’s near-miss.” (Female nurse, 5 years’ experience, KATH, Kumasi)

Others describe near-misses as being inherently unpredictable:

“…there are times you think… a baby is stable enough to leave but then suddenly it will die. Then there is somebody who is very ill, you think …tomorrow by the time you come in they will not be there. But he will be there and he will go home. So you can’t exactly say that you can distinguish between them.” (Female nurse, 20years’ experience, KBTH, Accra)

“We call them miracle babies.” (Male physician, 3 months’ experience, KATH, Kumasi)

Other providers suggested that whether or not a baby was a ‘near-miss’ may be a matter of opinion:

“It depends on how much effort is given to save the baby–one baby could be resuscitated and live and we call that a near-miss. Another baby could have arrived late, not gotten medicine in a timely way, and only later is it administered and the baby gets better. That could also be a near miss. I don’t think all of us will have the same point of view… It all depends on when you saw the baby (and) what you did to prevent the mortality.” (Female physician, 1 year experience, KATH, Kumasi)

When asked whether the classification of ‘near-miss’ was useful to clinical management, most providers (15 out of 18) indicated that they believed “near-miss” is a useful term.

“I think it’s even going to enhance that child’s survival rate, ‘cause you know that … child can stop breathing at any time and we need (a) bag and mask, and so you have your resuscitation tools right …(at) arm’s length or at a point where you don’t have to go searching, where is this, where is that? I think it’s going to provide and enhance the care of those ones who are near misses to prevent us from missing them actually.” (Female house officer, 1 month experience, KBTH, Accra)

“…The fact that we can classify a baby as a near miss needing a lot of attention can help us in our day-to-day work so that we…take care of those who really need our attention, you know, earlier than those who can actually wait a bit.”(Female physician, 1 year experience, KBTH, Accra)

But 3 of the 18 respondents said “near miss” was not a useful term because all babies in the NICU require attention and vigilance, and favoring some over others may not be ideal.

“If you are thinking somebody is very stable, so perhaps you are giving your attention to the person you think is so ill, and then only for you to realize that the other person who…you thought was so stable rather turns bad and just dies…. So I don’t think it’s good for you to start distinguishing that this particular baby is ok, so let me turn all my attention to this one.” (Female nurse, 20 years experience, KBTH, Accra)

“I don’t think putting a label on them will make a difference.” (Male physician, 3 months’ experience, KATH, Kumasi)

Nonetheless, 16 out of 18 respondents said that the classification of “near-miss” might change how they managed the baby.

“…If it’s a near miss, then you know that … there can be consequences so we have to look out for them.” (Female physician, 1 year experience, KATH, Kumasi)

## Discussion

This study suggests that few providers in Ghanaian NICUs had heard of neonatal near-misses or were familiar with what might constitute a near-miss. Most agreed that babies who survive a life-threatening event would warrant additional attention. While many providers expressed support for the use of a near-miss classification system to help them identify the newborns at greatest risk of dying shortly after birth, a vocal minority of providers felt that all newborns in a NICU have the potential to take an abrupt turn for the worse, and thus a near-miss classification may provide a false sense of security. Providers expressed concern that resources ought not be diverted for only a subset of newborns in the NICU.

The findings of this study raise the question of whether a near-miss classification–which some posit is a retrospective diagnosis for cases that are identical to deaths in all but the outcome, which can only be determined in hindsight[7]–is relevant in a clinical care provision setting or ought to be limited to use as a quality indicator. To date, most assessments of near-misses have been conducted retrospectively on large datasets to determine the differences between neonatal death rates and the rates of near-misses, or to explore the use of near-misses as a proxy for quality of care.[5,6,14,15] Yet data presented here suggest that some clinicians would find it useful to have a list of criteria that indicate a baby might qualify as a near-miss. This may be most appropriate for lower level providers who may not have the depth of experience to understand that certain indicators are more severe than others and that babies with such indicators require additional monitoring and vigilance. While some providers might argue that such criteria ought to be obvious, the quality assurance literature is rife with examples where codifying and creating checklists of the obvious can lead to enormous quality improvements.[16] Thus it is entirely possible that the creation and implementation of a neonatal near-miss classification system could help identify and correct weaknesses in facility-based newborn care. It is also possible that a near-miss classification system could serve as a reminder to engage in longer-term monitoring and follow-up among those newborns who survived a life-threatening complication. Such follow-up and monitoring is important not only for providers, but also for parents and primary care givers, who can be reminded to bring the baby back for periodic examinations to identify any potential problems before they become severe.

One challenge with assessing neonatal near-misses is the difficulty in developing criteria that works for all settings, amid the wide variability of clinical, interventional and laboratory markers that have been considered as newborn scoring tools or neonatal near miss tools. These criteria would have to be simple, feasible to assess, meaningful for clinicians, managers and health care professionals, stable in terms of severity, and applicable to a variety of settings.[17] Current research by Kale and colleagues suggests that ‘pragmatic criteria’ (birthweight, gestational age, 5’ Apgar score) can be effectively used to define neonatal near-misses.[18] Such simple criteria allow for comparisons across varying socioeconomic situations and can be applied in the absence of technologically advanced screening and treatment modalities. [18]

This study has several notable strengths. First, it took place across three tertiary care centers (all University hospitals) in different regions of Ghana, and it included health care providers of varying ages, backgrounds, and levels of experience in a NICU setting. Thus the findings reflect an extremely diverse group of participants, yet all work in high acuity settings with greater-than-average exposure to emerging trends in neonatal care as a function of being based at University teaching hospitals. Nonetheless, results were consistent: providers were not familiar with the concept of neonatal near-misses. In addition, this study used a semi-structured interview format with very little prompting, allowing providers to speak in their own words about how they perceived near-misses. This methodology was intentional: We did not want to bias responses through the use of structured questionnaires with multiple choice answers that might provide clues to how the authors conceptualized near-misses before respondents had a chance to provide their own opinions. Finally, this is the first study that we know of that asks providers what they think of the term ‘near-miss’ in the context of ill newborns in a resource-limited setting.

Despite its strengths, this study’s qualitative methodology precludes generalization of the findings. Designed as an exploratory study, this research aimed to further our understanding of provider perceptions. Thus further research is warranted on the construct of ‘near-misses’ in a low resource setting. In particular, it is not clear whether the categorization of ‘near-miss’ is appropriate in low-resource NICUs, given that most babies who are admitted are extremely ill and may all classify as near-misses. Another limitation of this study relates to the differences in experiences between the doctors interviewed (who had fewer years of experience both overall and in the NICU) and the nurses. We attribute this to the greater likelihood of more junior physicians spending more time at the hospital, while more senior physicians were less likely to be on-site and perceived as available for interviews. Nonetheless, our results may be different if more senior-level providers were included in our sample.

## Conclusions

This study explored the perceptions of healthcare providers with varying levels of experience in Ghana on the concept of a “neonatal near miss”. The majority of providers were relatively unfamiliar with the concept, although most acknowledged a baby who survived a life-threatening event warranted additional attention, thus agreeing a neonatal near-miss classification would be useful. However, further research is warranted to determine the practical implications of using a near miss tool in the process of providing care in a resource-limited setting and whether it might be best reserved as a retrospective indicator of overall quality of care provided.
